# Phenotype and genotype of  lactic acid bacteria (LAB) isolated from the tiger grouper *Epinephelus fuscoguttatus* alimentary tract

**DOI:** 10.12688/f1000research.12734.1

**Published:** 2017-11-10

**Authors:** Nursyirwani Nursyirwani, Widya Asmara, Agnesia Endang Tri Hastuti Wahyuni, Triyanto Triyanto, Muhammad Fauzi, Zainal Abidin Muchlisin

**Affiliations:** 1 Faculty of Fishery and Marine Science, Universitas Riau, Pekanbaru, Indonesia; 2 Faculty of Veterinary Medicine, Gadjah Mada University, Yogyakarta, Indonesia; 3 Department of Fisheries, Faculty of Agriculture, Gadjah Mada University, Yogyakarta, Indonesia; 4 Faculty of Marine and Fisheries, Syiah Kuala University, Banda Aceh, Indonesia

**Keywords:** phenotype, genotype, characterization, lactic acid bacteria, tiger grouper

## Abstract

Lactic acid bacteria (LAB) have been isolated successfully from the tiger grouper *Epinephelus* *fuscoguttatus* intestine. However, their genus or species have not been identified. Therefore, the objective of the present study was to characterize the three isolated LAB (KSBU-12C, KSBU-5Da, and KSBU-9) based on their phenotype and genotype. The LAB phenotype was examined by observing morphological features including cell morphology, spore production and motility. The physiological tests were performed in 6.5% NaCl at the  temperatures of 10 ^o^C and 45 ^o^C, and the biochemical tests were evaluated based on the production of enzymes catalase, oxidase and arginine dehydrolase, following  the Standard Analytical Profile Index, API 50 CH kit.  The genotype was examined based on 16S rDNA gene sequence analysis , and the products were analyzed using the BLAST (Basic Local Alignment Search Tool) NCBI database. The three isolates (KSBU-5Da, KSBU-12C, and KSBU-9) were categorized into the genus *Enterococcus*. 16S rDNA sequence analysis indicated that the isolates had 99% similarity to *E. hirae* ATCC 9790, registered in GenBank with accession number NR_075022.1. It was concluded that the three LAB isolates taken from the tiger grouper *Epinephelus fuscoguttatus* are *E. hirae*.

## Introduction

Lactic acid bacteria (LAB) have been investigated as probiotics in the feed of terrestrial and aquatic farmed animals ^1– 7^. This is based on the fact that LAB inhabit the human and animal gastrointestinal tract. This group of bacteria also convert lactose to acetic acid, thus decreasing gastrointestinal pH and naturally preventing colonization of harmful bacteria ^8^. This is because they have the ability to produce the inhibitory materials that inhibit pathogen growth in the digestive tract ^9^. Besides that, the probiotics can stimulate host immunity responses ^2, 9, 10^.

LAB are a group of gram-positive, non-sporulating bacteria, in cocci or rod form. They can live in aerobic or facultative anaerobic conditions. LAB are able to produce lactic acid through the fermentation process of carbohydrates ^11, 12^, and the optimum growth is at pH 5.5 to 6.0, so growth is restricted to neutral and alkaline conditions ^13^. Those naturally occurring bacteria are non-pathogenic to humans and animals, hence being generally recognized as safe (GRAS) organisms ^14^.

According to Parada *et al.* ^15^ and Carr *et al.* ^16^ 17l generas of LAB have been described worldwide. They are common in food products, for instance milk, meat, fruits, and vegetables, and also in genital and alimentary tracts of humans and animals ^17^, including fish intestines ^18, 19^.

Molecular approaches based on DNA such as 16S rRNA sequencing have been used to characterize the intestinal microflora, and this method is proven to be accurate and fast ^20^. For instance, Balcazar *et al.* ^21^ have identified LAB isolates from salmonid fish using 16S rRNA genes, and they found that there were 6 species of LAB, namely *Lactobacillus curvatus, L. sakei, L. plantarum, Lactococcus lactis* subsp. *cremoris*, *L. lactis* subsp. *lactis*, *Carnobacterium maltaromaticum,* and *Leuconostoc mesenteroides*. Sun *et al.* ^22^ investigated microbiota from gastrointestinal tracts of the orange-spotted grouper *Epinephelus coioides*, using a standard isolation procedure in combination with analysis of 16S rRNA sequences, and found several bacterial species, namely *Vibrio parahaemolyticus*, *V. harveyi*, *V. metschnikovi*, *V. alginolyticus*, *Delftia acidovorans, Pseudomonas putida, Acinetobacter baumannii*, *Burkholderia cepacia*, *Erwinia carotovora*, *S. aureus*, *L. lactis*, *L. casei*, *E. faecium*, *Bacillus pumilus*, *B. clausii,* and *Psychrobacter* sp.

Nursyirwani *et al.* ^23^ successfully isolated three LAB from the tiger grouper alimentary tract, and found that LAB have antibacterial activity against *Vibrio alginolyticus*. However, the taxonomic classification of these bacteria has not been examined. Therefore, the objective of the present study was to characterize the three LAB isolates from tiger grouper based on their phenotype and genotype.

## Methods

### Collection of LAB isolates

The experiment was carried out in compliance with the ethical guidelines provided by the Research Institution of Riau University (SOP/02/PL/LPPM/2016). Lactic acid bacteria (LAB) had been isolated from the tiger grouper ( *E. fuscoguttatus*) alimentary tract following the procedure of Bucio *et al* ^24^. The tiger grouper *Epinephelus fuscoguttatus* was taken from the aquaculture pond in Situbondo of East Java, Indonesia. The alimentary tract was removed from the fish, opened, and the gut content was removed. Then, the internal wall of the tract was scraped gently, and the mucus was collected in a sterile test tube containing 9 ml of phosphate buffer saline (PBS) solution at pH 7.2. The suspension was serial in PBS solution from 10 ^-1^ to 10 ^-6^. A volume of 0.1 ml of each serial dilution was inoculated in petri-dish containing deMan-Rogasa-Sharpe (MRS, Oxoid) agar medium. All inoculated dishes were incubated at 37°C for 24–48 hours. The bacterial colonies were reinoculated in new fresh MRS agar medium. Then, the grown colonies were inoculated on medium agar of glucose-yeast extract-pepton, supplemented with CaCO _3_ (GYP+CaCO _3_) agar and incubated at 37°C for 24 hours. The presence of LAB was indicated by the growth of white colonies surrounded by clear zones. The isolates were then examined for gram staining and catalase tests. Gram-positive and negative catalase isolates were selected and reinoculated in new fresh MRS agar and incubated at 37°C for 24 hours. The isolates were stored in a refrigerator at a temperature of -10°C before being used for the next tests.

### Bacterial isolates

The three LAB isolates (KSBU-12C, KSBU-5Da, and KSBU-9) previously isolated from the intestine of the tiger grouper were reinoculated in MRS broth (MRS, Merck). Phenotype characterization was based on cell morphology, physiology, and biochemical tests. Cell morphology involves observing the shape of the cells, spore production, and motility. Physiological tests were performed in 6.5% NaCl at 10°C and 45°C. Biochemical tests were based on the production of enzymes catalase, oxidase, and arginine dehydrolase following the Standard Analytical Profile Index, API 50 CH kit (BioMerieux SA, Marcy I’Etoil, France). Molecular characterization of 16S rDNA gene sequences allowed identification of the genotype of the LAB isolates.

### DNA extraction

DNA of isolates KSBU-12C, KSBU-5Da, and KSBU-9 was extracted following the procedures of Ausubel *et al* ^25^. Each of the isolates were reinoculated in 5ml of MRS broth and then incubated at 30°C for 48 hours. 

The 1.5 ml culture was centrifuged at 13000 rpm for 2 minutes, the supernatant was discarded, the pellet was resuspended in 467 µL of TE buffer, and 30 µL of lysozyme was added and then incubated at 37°C for 30 minutes. The suspension was added to 30 µL of 10% SDS and mixed with 3 µL of K proteinase and incubated at 37°C for an hour. The mixture was added to 100 µL 5 M NaCl, mixed well, added to 80 µL of CTAB/NaCl solution, mixed thoroughly, and incubated for 10 minutes at 65°C. The mixture was further incubated at 65°C for 10 minutes, mixed by stirring up and down 100 times, and the same volume (0.7–0.8 ml) of phenol/chloroform/isoamyl-alcohol (PCIAA) was added, mixed well, and centrifuged for 5 minutes. The supernatant beneath the interphase was transferred to a new microtube, mixed with an equal volume of PCIAA, and centrifuged for 5 minutes.

The supernatant was placed into a new the microtube, then the isopropanol (60% of the volume) was added to the tube and centrifuged for two minutes. The supernatant was discarded, the DNA pellet was washed with 70% ethanol (±50 µL), centrifuged for 5 minutes, the ethanol was discarded, and the pellet was dried for 1 hour. Finally, the pellet was added to 100 µL TE buffer, vortexed, and stored at -20°C until it was used for further experiments.

### Amplification of 16S rDNA

The genome of bacterial 16S rDNA was amplified by PCR using a thermal cycler (Eppendorf, Mastercycler Personal). The PCR reaction consisted of 25 µL of final solution of 12.5 µL PCR mix (Promega), 1.0 µL of universal primer (1 ^st^ base) 24F (5-‘AGAG TTT GAT CCT GGC T-3’) and 1.0 µL primer 1540R (5-‘AAG G AGGT GAT CC AG CC GCA-3’), 0.5 µL of DNA template, and 10.0 µL of dH _2_O.

The thermocycler program was run with the following conditions: pre-denaturation at 94°C for 4 minutes; 29 cycles of denaturation at 94°C for 3 minutes; annealing at 55°C for 1 minute; extension at 72°C for 1 minute and 30 seconds; and the final extension at 72°C for 10 minutes. The PCR product was detected by running electrophoresis in 1% of agarose gel, stained with ethidium bromide (1 µL/10 ml), and visualized under a UV lamp for 1500 base pairs of the target product.

### DNA sequence analysis

The PCR product with clear bands was sent to 1 ^st^ Base Laboratories in Singapore for sequencing. The sequencing program was performed by ABI PRISM 3730×L GENETIC ANALYZER (Applied Biosystems, USA). The sequenced products were blasted (NCBI Basic Local Alignment Search Tool) ^26^, and the results were presented as homology (%) of bacterial DNA sequences to the database sequences ^27^.

## Results

The three LAB isolates were categorized as cocci, gram-positive, non-motile, and non-spore forming. The LAB did not produce catalase, oxidase, and arginine dehydrolase, and were facultative anaerobes, with growth at 45°C at concentrations of 6.5% NaCl. However, differences were observed in the ability to produce acid from carbohydrates provided in the API 50 CH kit. Acid was not produced from substrates _L_-arabinose, _D_-raffinose, and _D_-xylose by the KSBU-12C isolate, meanwhile the KSBU-5Da isolate did not use _L_-arabinose, gluconate, glycerol, _D_-mannose, sorbitol, _D_-tagatose, and _D_-xylose; and the KSBU-9 isolate used _L_-arabinose, and _D_-xylose. The cell morphology, and the physiological and biochemical characteristics of the isolates are presented in detail in Table 1.

**Table 1.** Phenotype of KSBU-12C, KSBU-5Da, and KSBU-9 isolates in comparison to *Enterococcus* sp. ^28^.

Phenotype characters	KSBU-12C	KSBU-5Da	KSBU-9	Group and species of *Enterococcus*		
				*E. faecium*	*E. durans*	*E. hirae*
**Cell morphology**						
Shape	Coccus	Coccus	Coccus	Coccus	Coccus	Coccus
Gram staining	+	+	+	+	+	+
Spore formation	-	-	-	-	-	-
Motility	-	-	-	-	-	-
Use of pyruvate	n.a	n.a	n.a	-	-	-
**Production of:**						
Catalase	-	-	-	-	-	-
Oxidase	-	-	-	-	-	-
Alkaline phosphatase	n.a	n.a	n.a	-	-	-
Arginine dehydrolase	-	-	-	+	+	+
Pyrrolidonyl arylamidase	n.a	n.a	n.a	+	+	+
O/F	O/F	O/F	O/F	n.a	n.a	n.a
**Growth at:**						
10°C	n.a	n.a	n.a	+	+	+
45°C	+	+	+	+	+	+
NaCl 6.5%	+	+	+	+	+	+
Oxygen requirement	Anaerobic facultative	Anaerobic facultative	Anaerobic facultative	Anaerobic facultative	Anaerobic facultative	Anaerobic facultative
**Production of :**						
Acetoin	n.a	n.a	n.a	+	+	+
Antigen group D	n.a	n.a	n.a	+	+	+
Leucine arylamidase	n.a	n.a	n.a	+	+	+
α-Galactosidase	n.a	n.a	n.a	-	-	+
β-Galactosidase	n.a	n.a	n.a	+	D	+
β-Glucuronidase	n.a	n.a	n.a	-	-	-
**Hydrolysis of:**						
Esculin	+	+	+	+	+	+
Hippurate	n.a	n.a	n.a	d	D	D
Starch	n.a	n.a	n.a	-		D
**Acid from:**						
Adonitol	-	-	-	-	-	-
D-Arabitol	-	-	-	-	-	-
Inulin	-	-	-	-	-	-
Melezitose	+	+	+	-	-	D
Ribose	+	+	+	+	+	+
_L_-Sorbose	-	-	-	-	-	-
*N*-Acetylglucosamine	+	+	+	+	+	+
Amygdalin	+	+	+	+	+	+
_D_-Arabinose	-	-	-	-	-	-
_L_-Arabinose	-	-	+	+	-	-
_L_-Arabitol	-	-	-	-	-	-
Arbutin	+	+	+	+	+	+
Cellobiose	+	+	+	+	+	+
Dulcitol	-	-	-	-	-	-
Erythritol	-	-	-	-	-	-
_D_-Fructose	+	+	+	+	+	+
_D_-Fucose	-	-	-	-	-	-
_L_-Fucose	-	-	-	-	-	-
Galactose	+	+	+	+	+	+
β-Gentiobiose	+	+	+	+	+	+
Gluconate	+	-	+	d	-	-
_D_-Glucose	+	+	+	+	+	+
Glycerol	+	-	+	d	-	d
Glycogen	-	-	-	-	-	-
Inositol	-	-	-	-	-	-
2-Keto-gluconate	-	-	-	-	-	-
5-Keto-gluconate	-	-	-	-	-	-
Lactose	+	+	+	+	+	+
_D_-Lyxose	-	-	-	-	-	-
Maltose	+	+	+	+	+	+
Mannitol	+	+	+	d	-	-
_D_-Mannose	+	-	+	+	+	+
Melibiose	+	+	+	d	D	d
α-Methyl-D-glucoside	-	-	-	-	-	-
α-Methyl-D-mannose	-	-	-	d	-	-
_D_-Raffinose	-	+	+	d	D	d
Rhamnose	-	-	-	d	-	-
Sorbitol	+	-	+	d	-	-
Starch (Amidon)	-	-	-	-	-	d
Sucrose	+	+	+	d	D	d
_D_-Tagatose	+	-	+	d	-	d
Trehalose	+	+	+	+	+	+
_D_-Turanose	-	-	-	-	-	d
Xylitol	-	-	-	-	-	-
_D_-Xylose	-	-	+	d	-	-
_L_-Xylose	-	-	-	-	-	-

Note: +, 90% or most strains are positive; -, 90% or most data are negative; O/F, oxidative or fermentative; nd, data not available; d, variable.

**Figure 1.**
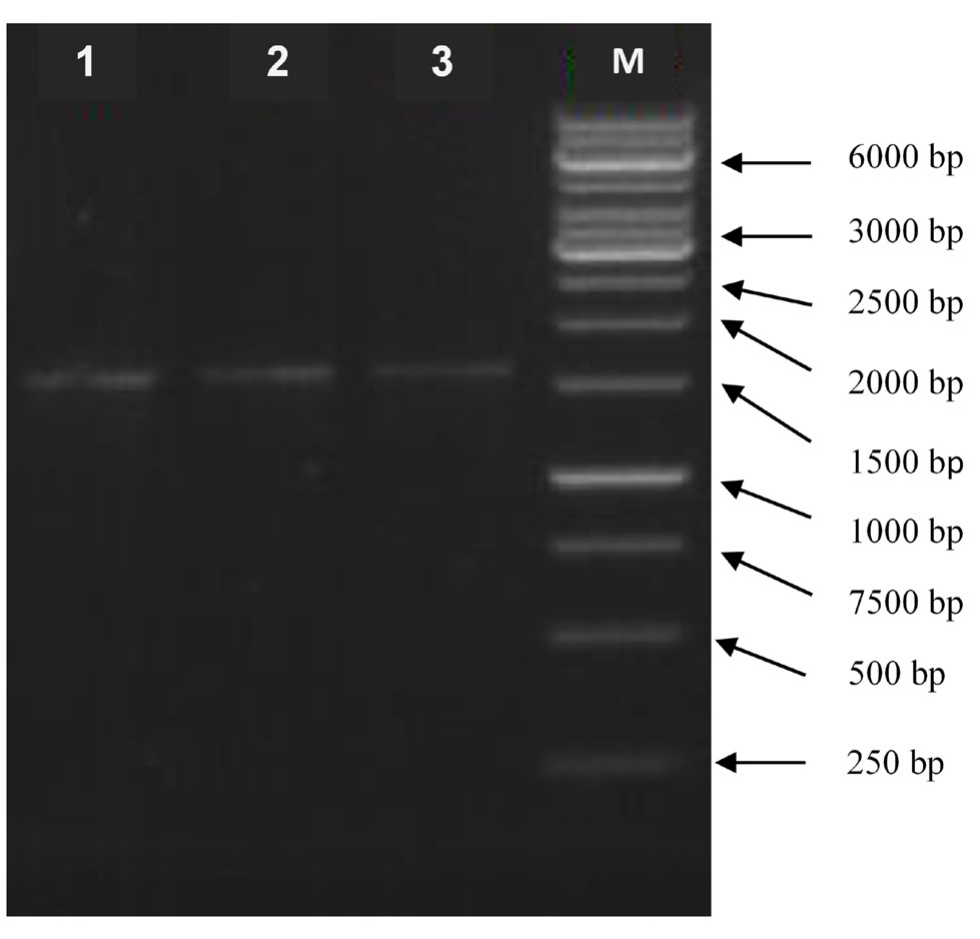
Purification product of 16S rDNA LAB genome of (1) KSBU-5Da; (2) KSBU-9; (3) KSBU-12C isolates; (4) marker DNA ladder ( *bp*).

**Figure 2.**
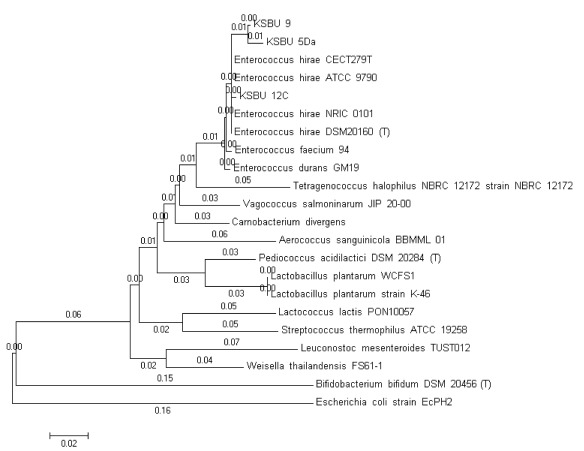
Phylogenetic tree constructed by the neighbor-joining method based on partial sequence of 16S rRNA gene for KSBU-12C, KSBU-5Da, and KSBU-9 isolated from tiger grouper. Scale 0.02 indicates sequence difference percentage. *Escherichia coli* strain EcPH2 was used as the outer group.

The 16S rRNA derived from the LAB isolates were amplified by PCR with two universal primers 24F (5-‘AGAG TTT GAT CCT GGC T-3’) and 1540R (5-‘AAG G AGGT GAT CC AG CC GCA-3’). The LAB isolates have a highly similar 16S rRNA sequence (99% similarity as shown in Table 2; those were 1510-bp for KSBU-12C and 1526-bp for KSBU-5Da and KSBU-9) ( Figure 1). A phylogenetic tree was constructed for the LAB isolates sequences using Clustal W, followed by the Mega 5 neighbor-joining program as shown in Figure 2.

**Table 2.** BLAST results of LAB isolates KSBU-12C, KSBU-5Da, and KSBU-9.

No.	Isolate code	BLAST results		Homology (%)
		Isolate name	*Accession* *number*	
1.	KSB-U 5Da	*Enterococcus hirae* ATCC 9790 *Enterococcus hirae* NRIC 0101 *Enterococcus hirae* C17456	NR 075022.1 AB362590.1 AY550918.1	99 99 99
2.	KSBU-12C	*Enterococcus hirae* ATCC 9790 *Enterococcus* sp. T5R2C10 *Enterococcus hirae* LMG 6399	NR 075022.1 JX193632.1 AJ301834.1	99 99 99
3.	KSBU-9	*Enterococcus hirae* ATCC 9790 *Enterococcus hirae* NRIC 0101 *Enterococcus hirae* CECT 279T	NR 075022.1 AB362590.1 AJ420799.1	99 99 99

## Discussion

Based on morphological, physiological, and biochemical characteristics, the LAB isolates (KSBU-12C, KSBU-5Da, and KSBU-9) show similarity to three bacterial species of the *Enterococcus* genus found by Ludwig *et al.* ^28^. The analysis of the 16S rDNA sequence indicated that KSBU-5Da, KSBU-12C, and KSBU-9 isolates were of the *Enterococcus* genus, and with high similarity to *E. hirae* ATCC-9790 registered in GeneBank with accession number NR_075022.1 ( Table 2). Each of the LAB isolates indicated 99% homology with *E. hirae* ATCC 9790. Strains of bacteria with the same or more than 97% 16S rRNA gene sequence belonged to one species ^29^.

*E. hirae* ATCC 9790 is a gram-positive LAB used as a model organism in basic research for four decades ^30^. *E. hirae* was first isolated in the intestines of pigs and chickens ^31^. *E. hirae* strain NRIC 0101 was collected by the Japan Nodai Culture Collection Center, NCCC ^32^. In addition, *E*. *hirae* C17456 was a species isolated from chickens ^33^. In summary *, Enterococcus hirae* has been found in humans, poultry, foods for instant dosa batter, and in the environment ^34– 37^. Together with *E. faecium, E. faecalis, E.casseliflavus,* and *E. mundtii* they are enterococci which were predominant in coastal waters and sediments of South of California ^38^. *E. hirae* was one of the homofermentative strains obtained from traditional fermentation products such as pla-som and pla-chom (fermented fish) in Thailand ^39^. It has been shown that *E. hirae* K34, *L. plantarum* K39, and *L. plantarum* K50 isolated from *Kung-Som* (fermentative shrimp) display antimicrobial activity against pathogenic bacterial strains of *Bacillus cereus*, *E. coli*, *Staphylococcus aureus*, *Salmonella typhimurium*, *Vibrio cholerae,* and *Listeria monocytogenes* ^40^.

Information on the use of *E. hirae* as a probiotic in aquaculture is still limited. Mazurkiewicz *et al.* ^41^ reported that *E. hirae* isolated from the intestine of common carp ( *Cyprinus carpio* L.) and applied into feed did not have a significant effect on the growth performance of the common carp. In contrary, another report by Adnan *et al.* ^42^ and Carlos *et al.* ^43^ showed that *E. hirae* from freshwater fish *Catla catla* and rainbow trout *Oncorhynchus mykiss* had a significant effect on inhibiting the growth of pathogens such as *Escherichia coli*, *Staphylococcus aureus*, *Salmonella typhi* and *Pseudomonas* spp.

In the present study, the role of *E. hirae* isolated from tiger grouper fish the growth performance of fish has not been evaluated yet. Therefore, further study will be needed to examine the effect of these bacteria on the feeding of cultured fish.

## Conclusions

This research has successfully characterized three of the LAB isolates (KSBU-12C, KSBU-5Da, and KSBU-9) based on their phenotype and genotype. All isolates were determined to be *E. hirae*.

## Data availability

The data referenced by this article are under copyright with the following copyright statement: Copyright: © 2017 Nursyirwani N et al.

Sequenced DNA of LAB isolates can be found in NCBI GenBank, with accession numbers MF977716 to MF977718.
