# Supplementary material for: Phenotype and genotype of  lactic acid bacteria (LAB) isolated from the tiger grouper Epinephelus fuscoguttatus alimentary tract
Source: F1000Res. 2017 Nov 10;6:1984. [Version 1] doi: 10.12688/f1000research.12734.1 (PMC5717476; doi:10.12688/f1000research.12734.1)
Supplement: Peer review history

# Peer review: Referee response for version 1

**DOI:** 10.5256/f1000research.13795.r28009

**Published:** 2017-12-04

**Authors:** Asus Maizar Suryanto Hertika

**Affiliations:** 1 Faculty of Fisheries and Marine Sciences, University of Brawijaya, Malang, Indonesia

Thank you, here are some comments about the contents of the article. 

1. In the background it has not been explained specifically why the characterization of lactic acid bacteria isolated from the grouper intestine. The question is that the bacteria will be mixed with fish feed in order to increase the digestibility of the food or the prevention of pathogens that attack groupers.
2. Procedure of taking bacterial inoculum in the grouper's bowel. Is the fish fasted first? This is to know the bacteria do live in the intestines of grouper fish instead of bacterial contaminants derived from food given. 
3. In the discussion section has not been submitted phenotype characteristics of three types of bacterial isolates LAB. These traits include color, colony shape, surface roughness of the colony and will be better when displayed images with given information.
4. The RNA sequence results also need to be displayed to make the right conclusions in the characterization process. It would be great if supported with comparative literature.
5. It would be great if the discussion section uses more literature to discuss especially the latest journals. 

I have read this submission. I believe that I have an appropriate level of expertise to confirm that it is of an acceptable scientific standard.

# Peer review: Referee response for version 1

**DOI:** 10.5256/f1000research.13795.r27851

**Published:** 2017-11-22

**Authors:** Delianis Pringgenies

**Affiliations:** 1 Department of Marine Science, Faculty of Fisheries and Marine Science, Diponegoro University, Semarang, Indonesia

This article has excellent relevance value to both the development of marine science and is suitable for publication. This article should be indexed when minor improvements have been made.

 The abstract shows that this research is interesting.

 How many isolation works? Of all isolation, three isolates were selected. The method used in the study should be listed on the abstract. Show keywords, keywords generally refer from general to specific, and not mentioned in title or abstract.

 Introduction: It needs to be rewritten more purposefully, including backgrounds on topic selection and importance. Writing in English should pay attention to diction and grammar.

 Method: It needs to include the reference method used. The exposure method has not been written in a systematic or accurate way, so it can be repeated by others.

 Results and Discussion: There are still repeated discussions. References used are not current, need to use the latest references.

 An annotated version of the Word document can be viewed here.

I have read this submission. I believe that I have an appropriate level of expertise to confirm that it is of an acceptable scientific standard.

# Peer review: Referee response for version 1

**DOI:** 10.5256/f1000research.13795.r27853

**Published:** 2017-11-14

**Authors:** Medana Zamfir

**Affiliations:** 1 Institute of Biology Bucharest of the Romanian Academy, Bucharest, Romania

The paper describes the identification of three LAB isolates from the alimentary tract of a tiger grouper. The identification was done using various phenotypic and genotypic methods.

 However, there are some remarks or suggestions that can be made:

 - First of all, the authors mention that LAB strains were previously isolated; why they included then in the Methods section and how they were isolated?; on the other hand, they do not mention from how many individuals they isolated these strains from? Were they isolated from one fish? Then, in fact, it is only one strain, since all three were identified in the end as Ent. hirae. 

 - Introduction:

- "According to Pareda et al. ... 17l genera..." - that "l" doesn't belong there.
- I think the authors should use "microbiota" instead of "microflora".

- Methods:

      - Collection of LAB isolates

- "The suspension was serial in PBS..." - I think "diluted" is missing.
- "Gram positive and negative catalase isolates..." should be "Gram positive and catalase negative isolates..."
- It is not clear how they kept the isolates, at -10 degrees, but on agar media?
- The subtitle "Bacterial isolates" should be "Identification of the bacterial isolates" since they are talking about identification.
- "Physiological tests were performed in 6.5% NaCl..." should be maybe "... in the presence of 6.5% NaCl..."
-  "... production of enzymes **such as**..."

      - Amplification of 16S rDNA

-  "The genome of bacterial 16S rDNA..." should be " The 16S rDNA gene from the bacterial genome..."

- Results:

- In Table 1 there is a discrepancy between the legend and the table. For instance, in the Table they have "n.a"., while in the legend is "n.d".; in the table they have both "d" and "D", and in the legend only "d". Moreover, there are many rows with data only for the Enterococcus species, but for the strains isolated by them, there are no data available. What is the point to show these things?
- Some information from the Methods is repeated in the Results, such as the primers used.
- The paragraph with the similarities among the sequences and 16S rDNA amplicons, with Fig. 1 and Table 2, is not very clear. Perhaps they should mention first that the amplicons obtained with the universal primers were very similar (and give their size and the reference to Fig. 1) and then they should mention about the high similarity among the sequences (99%) with the sequence of Ent. hirae (reference to Table 2).  

 

 - Discussion: 

- This section can be improved, maybe with information about the source of this strain in the alimentary tract, the importance for the host, why was the only strain found in their samples, etc.
-  There is a paragraph that I would suggest to be changed in: " Strains... with the same or more than 97% homology of the 16S rRNA gene sequence may be considered to belong to the same species".
- "Ent. hirae was first isolated..." - I think the word "from" is missing.     

I have read this submission. I believe that I have an appropriate level of expertise to confirm that it is of an acceptable scientific standard, however I have significant reservations, as outlined above.
